# Automatic Echographic Detection of Halloysite Clay Nanotubes in a Low Concentration Range

**DOI:** 10.3390/nano6040066

**Published:** 2016-04-11

**Authors:** Francesco Conversano, Paola Pisani, Ernesto Casciaro, Marco Di Paola, Stefano Leporatti, Roberto Franchini, Alessandra Quarta, Giuseppe Gigli, Sergio Casciaro

**Affiliations:** 1 National Research Council, Institute of Clinical Physiology, Lecce 73100, Italy; conversano@ifc.cnr.it (F.C.); pisanip@ifc.cnr.it (P.P.); ernesto.casciaro@ifc.cnr.it (E.C.); m.dipaola@ifc.cnr.it (M.D.P.); rfranchini@ifc.cnr.it (R.F.); 2 National Research Council, Institute of Nanotechnology, Lecce 73100, Italy; stefano.leporatti@nanotec.cnr.it (S.L.); alessandra.quarta@nanotec.cnr.it (A.Q.); giuseppe.gigli@nanotec.cnr.it (G.G.)

**Keywords:** ultrasound contrast agents, automatic nanoparticle detection, automatic tissue typing, halloysite clay nanotubes, cell targeting, nanoimaging

## Abstract

Aim of this work was to investigate the automatic echographic detection of an experimental drug delivery agent, halloysite clay nanotubes (HNTs), by employing an innovative method based on advanced spectral analysis of the corresponding “raw” radiofrequency backscatter signals. Different HNT concentrations in a low range (5.5–66 × 10^10^ part/mL, equivalent to 0.25–3.00 mg/mL) were dispersed in custom-designed tissue-mimicking phantoms and imaged through a clinically-available echographic device at a conventional ultrasound diagnostic frequency (10 MHz). The most effective response (sensitivity = 60%, specificity = 95%), was found at a concentration of 33 × 10^10^ part/mL (1.5 mg/mL), representing a kind of best compromise between the need of enough particles to introduce detectable spectral modifications in the backscattered signal and the necessity to avoid the losses of spectral peculiarity associated to higher HNT concentrations. Based on theoretical considerations and quantitative comparisons with literature-available results, this concentration could also represent an optimal concentration level for the automatic echographic detection of different solid nanoparticles when employing a similar ultrasound frequency. Future dedicated studies will assess the actual clinical usefulness of the proposed approach and the potential of HNTs for effective theranostic applications.

## 1. Introduction

Theranostics arises from the integration of diagnostic and therapeutic paths in a single treatment, whose potential benefits for patients can be maximized through the employment of smart nanoparticle-based agents, capable of performing controlled drug release in selected patient-specific target sites (“personalized medicine”) [1,2,3].

In this interesting emerging field, new nanosized ultrasound contrast agents (UCAs) are now playing a key role thanks to the rapid evolution of contrast-enhanced ultrasound technologies combined with bio-nanotechnologies, showing good perspectives for molecular imaging and targeted therapy applications [4]. In fact, last-generation nanosized UCAs overcome all the main limitations presented by conventional microbubble UCAs, including in particular the fact that microbubble employment is definitely restricted to intra-vascular applications [5,6], since, by exploiting the enhanced permeability and retention (EPR) effect, nanoparticles can easily extravasate to reach specific diseased cells located beyond the endovascular space [7,8,9,10,11].

At present, cellular and molecular imaging in the clinical routine is mainly confined to highly ionizing and expensive techniques such as positron emission tomography (PET). Currently, PET is the only technique capable of providing functional tissue information but, in order to obtain suitable anatomical information, it requires complementary scanning with computed tomography (CT) or magnetic resonance imaging (MRI), with augmented costs and, in the former case, an additional radiation dose as well.

Ultrasonic imaging is the most widely available medical imaging modality, owing to its safety, real-time features, and low price per examination [12]. However, at clinical diagnostic transmit frequencies, healthy and pathologic tissues normally do not present enough differences in the scattering of ultrasound waves to be effectively distinguished. Therefore, UCA-mediated image enhancement is often necessary to provide optimal contrast, reliable tissue discrimination and, possibly, objective tissue typing.

Novel experimental agents for targeted drug delivery have been recently tested on both cell cultures and living animals [13,14]. In this context, halloysite clay nanotubes (HNTs) represent a promising carrier for drug and gene delivery for several reasons [15,16,17,18]. Halloysite is a two-layered aluminosilicate clay, chemically similar to kaolin, characterized by a tubular shape and an empty lumen [19,20,21], capable of encapsulating, retaining, and slowly releasing active agents, and these abilities have been successfully tested on a wide range of pathological situations on both cellular and animal models [18,20,22,23,24,25,26,27,28]. HNTs are a natural product made of inexpensive materials compared to other nanotubes currently in use (e.g., carbon nanotubes [29,30]), with a simple manufacturing process and multiple chances of selective labeling through different chemistry of the inner and outer surfaces [26]. Thus, HNTs are good candidates for household materials and encapsulation of genes and drugs, they also present an ideal profile for cell targeting and possess excellent drug delivery features for several kinds of pathological cells, including cancer cells, together with an efficient intracellular delivery of siRNA for cell-specific gene silencing [18,20,22,23,24,25,26,27,28,31]. Additionally, both their good biocompatibility and very low cytotoxicity were demonstrated [17,32], and polymer-halloysite composites resulted suitable materials for medical implants, such as in bone repair, as well as suitable systems to isolate viable circulating tumor cells from blood [23,33,34,35,36].

In the last few years, new perspectives towards ultrasound molecular imaging at clinical frequencies employing commercially available echographic devices and different organic and inorganic nanoparticles or nanocomposites have been demonstrated, showing their diagnostic suitability as UCAs not only with conventional ultrasound imaging [37,38], but also with contrast-specific approaches such as harmonic imaging [39], multimodal imaging combining ultrasound and MRI [40], and dual-frequency image subtraction [41]. All of these studies [37,38,39,40,41] employed solid nanoparticles (typically silica nanospheres or various combinations of silica nanospheres with smaller superparamagnetic particles), since the use of solid nanoparticles as UCAs provides higher enhancement than liquid ones (because the difference in acoustic impedance between nanoparticles and the surrounding physiological environment is generally higher for solid nanoparticles than for liquid ones), and it also assures a prolonged stability with respect to nanobubbles (because bubble lifetime is limited by gas diffusion kinetics, which also affect bubble acoustic effectiveness, whereas solid particles do not suffer from such limitations) [37]. Regarding the employment HNTs as UCAs, they show properties similar to those of the other solid nanoparticles, with the additional advantages of being cheaper (since they are a natural product) and also intrinsically better suited for drug delivery purposes (thanks to their native tubular structure).

In two very recent studies [42,43] we preliminarily assessed the detectability of the ultrasound backscatter of HNTs in conventional echographic images obtained through a clinically-available diagnostic device. Overall, several possible configurations were tested on custom-designed tissue-mimicking phantoms, including in particular a wide range of diagnostic ultrasound frequencies (5.7–11 MHz) combined with HNT concentrations variable from 0.25 to 5.0 mg/mL.

Aim of the present study was to investigate the possibility of achieving an automatic detection of HNT-containing areas in the echographic images by employing an innovative method based on advanced spectral analysis of the corresponding “raw” radiofrequency (RF) backscatter signals. Taking into account the results of the referred works [42,43], and in order to develop the least invasive and safest possible approach, in this paper we focused our attention on the lower part of the previously considered HNT concentration range (0.25–3.0 mg/mL) combined with the employment of a 10-MHz ultrasound frequency, which was already shown to be the most suitable one in terms of ultrasound enhancement [42]. Furthermore, in order to facilitate the design of the next validation studies on cellular and animal models, we also performed a detailed physicochemical characterization of the adopted HNTs, which had not been addressed in the previous works.

## 2. Results and Discussion

### 2.1. Physicochemical Characterization of HNTs

**Figure 1:**
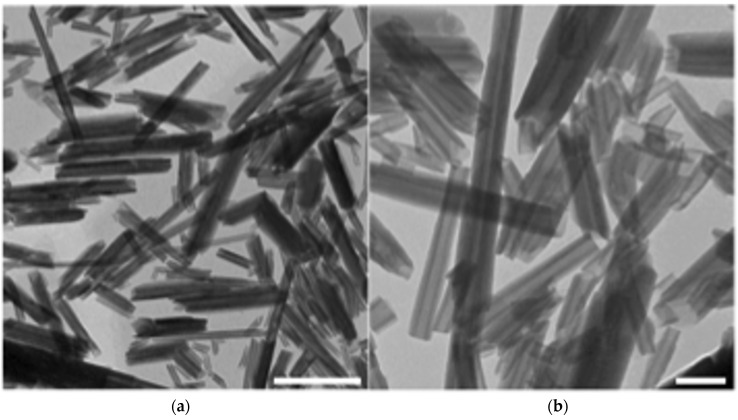
Typical transmission electron microscopy (TEM) images of halloysite clay nanotubes (HNTs): (**a**) panoramic image, showing the grade of polydispersity of HNT length (scale bar: 500 nm); and (**b**) high-magnification image, showing the hollow tubular structure of HNTs (scale bar: 100 nm).

Figure 1a,b show typical transmission electron microscopy (TEM) images, at different magnifications, obtained from a HNT solution spread on a flat support. Quantitative image analysis documented that most of the sample consists of cylindrical tubes, which measure from 40 to 60 nm in diameter and are somewhat polydispersed in length (range 0.5–1.5 μm). TEM images clearly show the empty lumen of the HNTs, from 10 to 20 nm in diameter (Figure 1b). For some tubes, we can also see loose packing of the outermost aluminosilicate sheets, which are not tightly rolled. In summary, the studied HNTs presented a length of 500–1500 nm, an external average diameter of 50 nm, and an average lumen diameter of about 15 nm.

**Figure 2:**
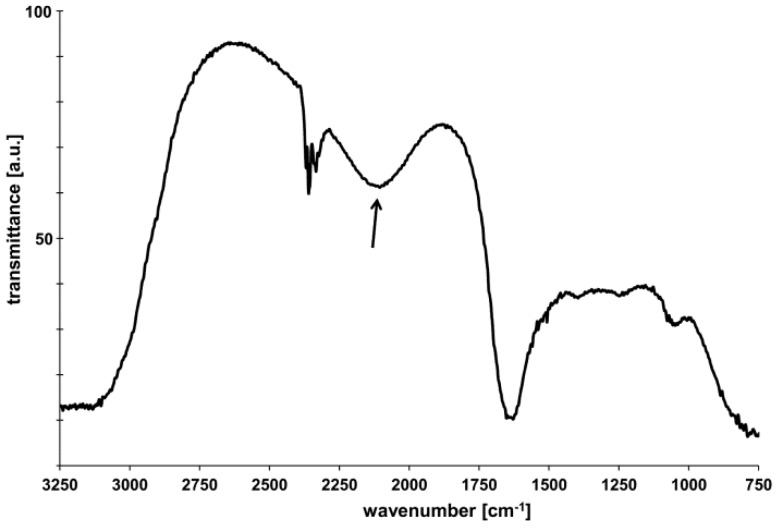
Fourier transform infrared (FT-IR) spectrum of HNTs: the arrow indicates the peak due to the Si–H signal (2120 cm^−1^).

As expected, the HNTs showed a negative Z-potential value (−50 mV) due to the hydroxylic groups on the surface of the nanotubes. The expected HNT structure was also confirmed by Fourier transform infrared (FT-IR) spectroscopy: the corresponding spectrum is reported in Figure 2 and is characterized by the typical Si–H signal detected at 2120 cm^−1^, as emphasized by the arrow in Figure 2.

**Figure 3:**
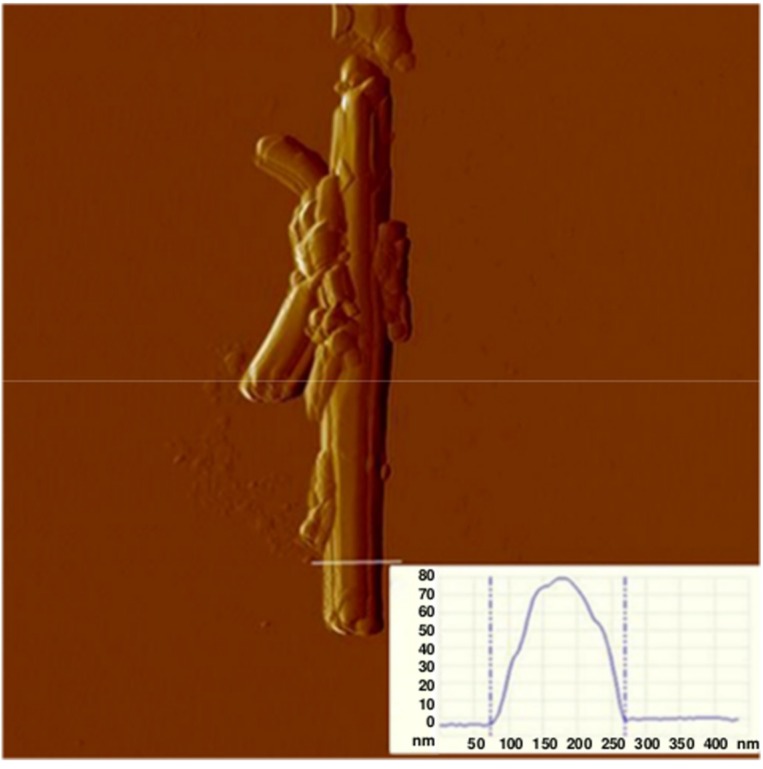
Scanning force microscopy (SFM) “tapping amplitude” image of a single HNT and corresponding section analysis (inset). (image size: 2.5 μm).

Scanning force microscopy (SFM) image (Figure 3) revealed the surface morphology of the HNTs, further demonstrating their rolled nature. From the SFM micrograph, which was acquired in “tapping mode” by measuring the tapping amplitude of an isolated HNT for enhanced morphological details. The tube diameter turned out to be approximately 80 nm (see the inset in Figure 2), which is larger than the one measured on the TEM images. This may be related to the SFM tip size convolution (the employed tip had a radius of curvature of about 20 nm) and to the presence of external loosely packed aluminosilicate layers, which are not well-resolved by TEM because they are only 1–2 nm thick but may be detected through SFM.

The features demonstrated through the performed physicochemical analyses clearly envisage HNT use as nanocarriers for high-efficiency cargo-loading. Hollow tubes, in fact, can easily encapsulate hydrophobic materials, such as natural antioxidants or drugs, as previously reported in the literature [18,20]. Furthermore, from TEM images it is also possible to appreciate their small apertures ranging from 10 to 20 nm, which allow stable loading of complex molecules, such as a single proteins [27]. In such a case, one of the lumens can also be chemically blocked in order to favor a much slower release. Altogether, these peculiar structural properties confirm the suitability of HNTs to be employed as a novel system for targeted delivery of therapeutic agents such as drugs and genes.

### 2.2. Automatic Echographic Detection of HNTs

**Figure 4:**
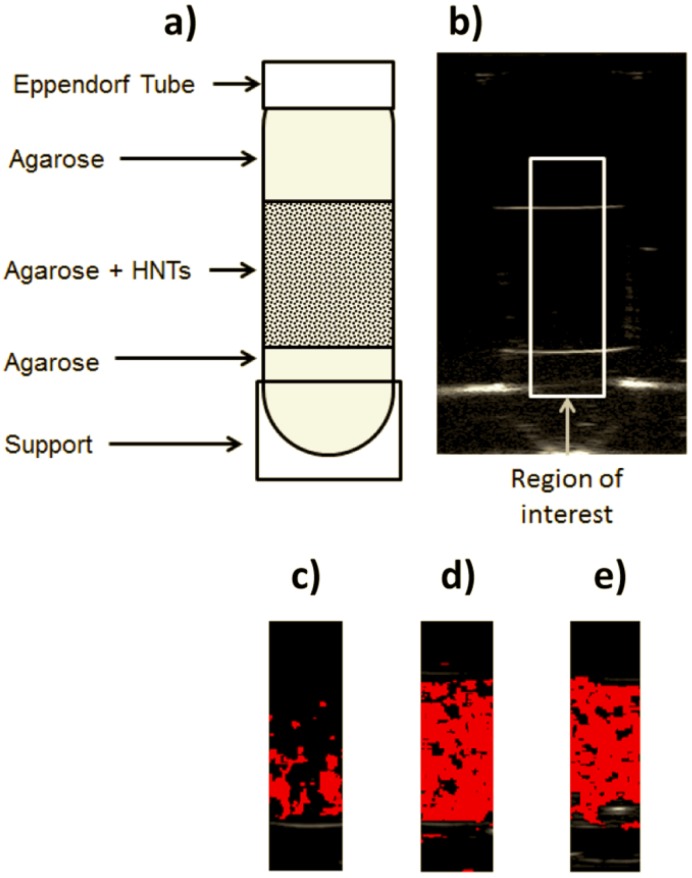
Automatic detection experiments: (**a**) scheme of the adopted phantom; (**b**) B-mode image of a control phantom (HNT concentration = 0 part/mL) with indication of the chosen region of interest (ROI); (**c**–**e**) sample images of the analyzed ROIs with the superimposed color maps for automatic HNT detection at the following concentrations: 16.5 × 10^10^ part/mL (**c**); 33 × 10^10^ part/mL (**d**); 66 × 10^10^ part/mL (**e**).

In the second part of the study we explored the possibility of exploiting the information introduced by the HNTs into the RF signal spectra for automatic detection purposes during a conventional echographic investigation. In particular, we illustrated how a tissue-mimicking layer containing such nanostructures placed between another two layers of pure tissue-mimicking material can be automatically and specifically detected in the conventional images. The results, which were obtained by applying a custom-developed configuration of the software RULES specifically optimized for HNT detection employing 10-MHz ultrasound pulses (see Section 3.2.3), are graphically summarized in Figure 4 for each considered HNT concentration.

**Figure 5:**
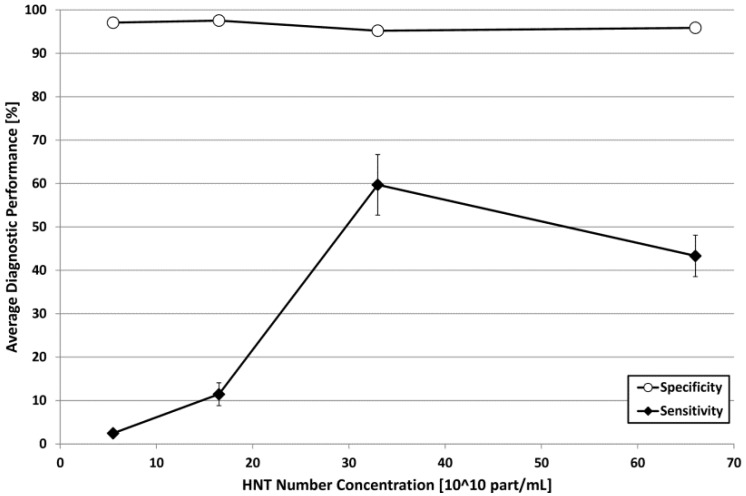
Diagnostic performance of the automatic HNT detection algorithm expressed through the plot of sensitivity and specificity as a function of HNT concentration. Error bars represent standard deviations, where visible.

In order to quantitatively evaluate the performance of the adopted approach, sensitivity and specificity were calculated according to Equations (1) and (2), obtaining for each concentration the average results illustrated in Figure 5.

Referring to Figure 5, specificity values were always above 95% (average value = 96.4%), but appeared somehow inversely dependent on the employed HNT concentration. Probably, the increased concentration level, which is typically accompanied by an augmented amplitude of backscatter signals in the case of solid nanoparticles [37,42], caused some reverberation effects that were misinterpreted by the software as the presence of HNTs in the phantom areas actually containing only pure agarose gel. However, at least in the considered experimental conditions, this effect was almost negligible and the specificity values were always in the range 95.2%–97.6%.

On the other hand, sensitivity showed a more complex trend, characterized by steep increments up to a HNT concentration of 33 × 10^10^ part/mL (sensitivity = 59.7%) followed by a visible decrease when the concentration was further doubled to 66 × 10^10^ part/mL (sensitivity = 43.3%). The corresponding values of Dice similarity coefficient (DSC), calculated according to Equation (3), showed the same trend, with the minimum in presence of 5.5 × 10^10^ HNTs/mL and the maximum in the case of 33 × 10^10^ HNTs/mL (Table 1).

This behavior can be due to the combination of two phenomena: (1) when the concentration of the particles in a given volume is doubled, the “mean free path” between two particles is decreased by about 20% and, beyond a certain limit, the interferences between the signals scattered by more particles in a limited space, although producing a higher backscatter amplitude, can result in a loss of spectral content peculiarities; and (2) as a consequence of the previous point, an augmented concentration statistically increases the likelihood that groups of two or more particles are so close to each other to be “seen” as a single bigger particle, actually decreasing the sample homogeneity and making more difficult the identification of a set of characterizing spectral features.

In the specific case of our phantoms, the HNT concentrations up to 16.5 × 10^10^ part/mL are probably too low to introduce clearly detectable contributions in the backscattered ultrasound spectra, whereas, for the above mentioned reasons, it could be also difficult to identify a characteristic “RF signature” in the presence of a HNT concentration of 66 × 10^10^ part/mL, which is likely to be too high for automatic detection through our adopted approach. Therefore, at least in presence of a 10-MHz ultrasound frequency, a concentration of 33 × 10^10^ part/mL could represent a kind of best compromise between the need of enough particles to introduce detectable spectral modifications in the backscattered signal and the necessity to avoid the losses of spectral peculiarity associated to higher concentrations.

In other words, the identified optimal HNT concentration can be considered as the most effective way to fill a target volume for automatic detection purposes in presence of a 10-MHz echographic investigation. This finding could also explain a previous empirical result of our research group on the automatic echographic detection of silica nanospheres of variable diameter (160, 330, and 660 nm) insonified at 7.5 MHz [37]: the fact that 330-nm nanospheres were detected with higher sensitivity with respect to both smaller and bigger particles at the same volume concentration, probably, could be explained by the fact that the number concentration of 330-nm nanospheres was very close to 33 × 10^10^ part/mL, while the number concentration resulted one order of magnitude higher for 160-nm nanospheres and one order of magnitude lower for 660-nm nanospheres. The employment of a similar ultrasound frequency could justify the fact that a similar number of nanoparticles for unit volume is necessary to obtain the most effective automatic detection.

Referring to the volume concentration of HNTs, our present results suggest that a concentration level of about 1.5 mg/mL (equivalent to 33 × 10^10^ part/mL) offers the maximum discrimination power in targeted tissues, being in particular automatically detectable with the highest sensitivity coupled with a very good specificity (95.2%). This further emphasizes the inopportunity of employing HNT concentrations higher than 1.5 mg/mL, which was already suggested by our preliminary studies [42], based on the experimental observation that the logarithmic compression of ultrasound backscatter amplitude that is performed by the echographic devices in the image reconstruction process makes negligible the image enhancement increments due to contrast doses higher than the identified threshold.

Actually, the described behavior of HNT detectability as a function of concentration could be in principle affected by possible phantom inhomogeneities, due to non-uniform dispersion of HNTs in the agarose gel. However, in the present study this phenomenon may have had only a slight influence, since, on the one hand, homogeneity of each prepared phantom was carefully checked through both visual and echographic inspections, and, on the other hand, the mentioned similarity with our previously obtained results on different nanoparticles in different phantoms [37] is unlikely to be just a coincidence and represents a further indirect confirmation of the robustness of present paper findings. Nevertheless, further validation studies will also provide quantitative information on the dispersion of HNTs in the agarose after jellification, by employing dedicated experimental techniques, such as refractometry.

### 2.3. Potential Applications of HNTs for Targeted Contrast Enhancement in Clinical Ultrasound Imaging

The application of ultrasound techniques in the emerging field of “molecular imaging”, which relies on the specific and selective detection of the molecular mechanisms of pathologic processes, has been very limited until a few years ago [37]. Then, the advent of targetable ultrasound contrast agents [44,45] introduced the possibility of “ultrasound molecular imaging”, based on the detection of acoustically active encapsulated gas microbubbles that are specifically retained in targeted diseased tissues.

Since microbubbles cannot leave the vascular space, they cannot reach cells located beyond the capillary vasculature, such as many cancer cells. Actually, tumor vessels have the tendency to be “leaky” [46] and to exhibit the so-called EPR effect, resulting in exaggerated extravasation and retention of particles that are smaller than the endothelium pore size, which is typically in the range 400–800 nm for tumor vasculature (while the corresponding common value for normal vessels is about 50 nm) [47].

In the present study we identified the value of 33 × 10^10^ part/mL as a possible ideal HNT concentration to be employed for ultrasound targeted imaging purposes at a conventional insonification frequency already employed in clinical routine (10 MHz). In fact, this specific concentration offers the key advantage of being automatically detectable on the common B-mode echographic images with high accuracy, thanks to the exploitation of the characteristic “signature” of HNTs in the corresponding RF signals (Figure 4 and Figure 5). In the case of HNTs, a number concentration of 33 × 10^10^ part/mL is equivalent to a dose of 1.5 mg/mL, whose safety to cells has to be carefully verified. Presently, we know from a very recent study [48] that, although uncoated HNTs show some cytotoxic effects already at a dose of 0.25 mg/mL, their surface coating with poly(ethylene glycol) (PEG) seems to make them completely non-toxic: this was verified on two different cell lines for HNT concentrations up to 0.5 mg/mL and incubation times up to 72 h. Further studies will be dedicated to specifically verify the biocompatibility of PEG-coated HNTs at doses up to 1.5 mg/mL and, in case, to the finer identification of an optimal and biocompatible concentration within the range 0.75–1.5 mg/mL.

Nevertheless, based on the findings discussed in the previous paragraph, a concentration close to 33 × 10^10^ part/mL is likely to represent the optimal contrast dose for automatic nanoparticle detection through an echographic investigation employing an ultrasound frequency close to 10 MHz, not only limited to HNTs, but also for other types of nanoparticles, for which the identified concentration level may be already safe and non-toxic to cells. On the other hand, different ideal concentration values could be determined for different ultrasound frequency ranges (*i.e.*, for clinical investigations of different organs).

The actual clinical usefulness of the proposed approach will be further verified through “*in vivo*” studies on animal models, but the results obtained in this study (*i.e.*, automatic HNT detection with specificity above 95% and sensitivity up to 60%) will provide a helpful guidance for subsequent parameter refinements through pre-clinical trials.

Moreover, as previously mentioned, since HNTs showed great promise in a range of applications such as creation of a nanoscale container for the encapsulation of biologically active molecules (e.g., biocides, enzymes, genes, and drugs), controlled drug delivery, and bioimplants [49], the effective localization of low HNT doses using a non-invasive, cheap, and widely available diagnostic imaging technique will be of crucial importance for improved planning of therapeutic strategies.

Finally, the future employment of specifically functionalized HNTs could also provide safe and cost-effective tissue characterization and therapeutic monitoring for an extensive range of pathologies, thus, possibly avoiding further diagnostic investigations based on ionizing radiation (*i.e.*, PET, currently still representing the gold standard technique for tumor molecular imaging), as well as many unnecessary invasive biopsies, surgical interventions, and aggressive medications.

## 3. Experimental Section

### 3.1. Physicochemical Characterization of HNTs

#### 3.1.1. Transmission Electron Microscopy

Purified dehydrated HNTs were obtained from Applied Minerals, Inc. (New York, NY, USA). TEM images were recorded on a Hitachi HD 2000 STEM (Tokyo, Japan) operated at an accelerating voltage of 80 kV. TEM samples were prepared by dropping a water-diluted solution of HNTs (0.25 mg/mL) on carbon-coated copper grids (Formvar/Carbon 300 Mesh Copper) and letting the solvent evaporate. The size of isolated HNTs in TEM images was measured by using the ImageJ software (National Institutes of Health, Bethesda, MD, USA).

#### 3.1.2. Z-Potential Measurements

Z-potential values were measured with a Zetasizer Nano ZS90 (Malvern, PA, USA), installing a 4.0 mW, 633 nm He–Ne laser. Measurements were made at 25 °C on aqueous solutions (pH = 7) of the HNTs. The values were determined using the Smoluchowski approximation (*f*(ka) = 1.5), and were estimated as the average of 20 repeated measurements.

#### 3.1.3. FT-IR Spectroscopy

FT-IR spectroscopy measurements were conducted in the 4000–400 cm^−1^ spectral range in transmission mode using a FT/IR-6300 type spectrophotometer (JASCO Inc., Easton, MD, USA): 1 mL of HNT solution at 0.1 mg/mL concentration was loaded onto the detector, while the corresponding baseline was obtained from ultrapure water.

#### 3.1.4. Scanning Force Microscopy

A SFM micrograph was obtained with Multimode-Picoforce (Veeco Instruments Inc., Santa Barbara, CA, USA) in air at room temperature “in tapping mode”, using TESPA cantilevers of 4 N/m spring constant. A drop of sample suspension was applied to a freshly-cleaved mica support. HNTs were studied after their dehydration. Experimental data were analyzed by NanoScope software (Version 7.30s1rs2, Bruker Nano Inc., Santa Barbara, CA, USA).

### 3.2. Echographic Acquisitions

#### 3.2.1. Preparation of HNT-Containing Tissue-Mimicking Phantoms

HNTs were dispersed in tissue-mimicking agarose gel samples, whose employment has been previously reported in literature as a suitable phantom configuration for “*in vitro*” studies of ultrasound signal enhancement produced by solid nanoparticles [9,37,50,51,52].

An essential parameter for phantom fabrication was the agarose concentration, since a too low agarose density would cause HNT sedimentation consequent to a slow jellification process, whereas a too high gel density, accompanied by a rapid jellification process, would result in the appearance of HNT aggregates. Preliminary studies focused on the specific HNT concentration range employed in the present work confirmed the suitability of the gel density value already employed in our previous papers (0.4% wt/vol) [42,43], which was then used for the preparation of both blank and HNT-containing gel samples.

Agarose powder (0.4 g/100 mL) was added to degassed water and the solution was heated to dissolve the agarose. The amount of 0.5 mL of this solution was then poured into a 2-mL Eppendorf tube. After 2 min in a freezer the agarose jelled, resulting in a layer of about 8 mm in thickness. In a separate vial, the desired amount of HNTs (see below) was added to the agarose solution and, after a 30 s sonication, 1 mL of this suspension was quickly poured into the Eppendorf tube, which was immediately placed in freezer to ensure a quick jelling preventing particle settlement. The obtained HNT-containing gel layer was about 15 mm in thickness and the phantom was finally completed by the addition of further 0.5 mL of pure agarose solution, resulting in the final structure illustrated in Figure 4a.

Four different HNT concentration levels were studied: 0.25, 0.75, 1.50 and 3.00 mg of HNTs in 1 mL of agarose gel. Considering the HNT dimensions observed in preliminary TEM experiments and their mass density (2.55 g/cm^3^), the adopted HNT concentration values were quantified in number of particles per milliliter and corresponded to 5.5, 16.5, 33, and 66 × 10^10^ part/mL. Three samples were obtained for each HNT concentration, including the controls at 0 part/mL, and all of them underwent the echographic scansion described in the next paragraph.

#### 3.2.2. Experimental Set-up

**Figure 6:**
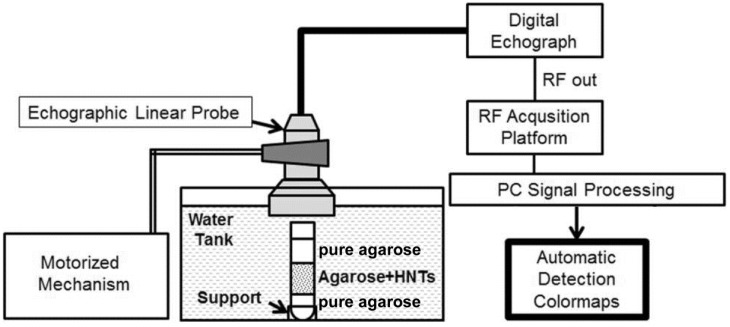
Scheme of the experimental data acquisition setup.

Echographic images and corresponding RF signals were acquired by means of the experimental set-up illustrated in Figure 6. A commercial echographic device, consisting of a clinically-available digital echograph (MyLab XVG, Esaote Spa, Genoa, Italy) was linked, by means of an optical fiber (1 Gb/s), to a research platform for the acquisition of RF signals digitized at 50 MHz, 16 bits (FEMMINA system, ELEN Spa, Florence, Italy) [53]. The echograph was equipped with a linear echographic transducer (LA523, Esaote Spa) mounted on the motorized mechanism of an infusion pump (KDS 100, KD Scientific Inc., Holliston, MA, USA) in order to perform automatic and repeatable scans of the phantoms. Echographic parameters were set to the following values: power = 50%, gain = 0 dB, time gain compensation (TGC) = linear (1 dB/cm), ultrasound frequency = 10 MHz, focus = 2 cm. The echographic transducer, partially immersed in water, was positioned perpendicular to the phantom surface at such a distance that the ultrasound beam focus was located half-way through the HNT-containing layer depth. For each analyzed phantom, 850 frames of RF data (152 tracks × 2980 points/track) were recorded at a constant frame-rate (12 fps), while the motorized mechanism moved the transducer at a constant scan speed (12.5 mm/min).

#### 3.2.3. RF Signal Analysis for Automatic HNT Detection

Raw RF signals acquired through FEMMINA were processed offline with a software tool for wavelet decomposition and spectral analyses (RULES, ELEN Spa) [54], in order to find a specific algorithm configuration to selectively discriminate the HNT-containing layer from the pure tissue-mimicking layers through the implementation of a dedicated color map superimposed on the grey-scale echographic images.

A 300-frame dataset corresponding to the central part of the Eppendorf tube was selected for each 850-frame acquisition and used for the following calculations.

The statistical evaluation of each selected RF data frame was conducted through a rectangular analysis window that was six tracks wide and 70 pixels high, corresponding to a spatial resolution of approximately 1.5 mm. The analysis window was translated over the frame, track by track in the horizontal direction and pixel by pixel in the vertical direction, extracting at each step the spectral characteristics of the underlying RF signal portions, which were provided as the numerical values of 25 spectral parameters.

The final output of this algorithm was the superimposition on the conventional B-mode images of a specific color map, identifying the presence of HNTs in each single frame. Sequences of color-mapped images were then analyzed through a dedicated software algorithm realized in MatLab 7.0 (The MathWorks, Natick, MA, USA), capable of counting the number of colored pixels in each frame, distinguishing between “true positives” (TP, pixels actually belonging to the HNT-containing layer) and “false positives” (FP, pixels not belonging to the target layer). In this way, for each frame, it was possible to calculate sensitivity and specificity of the detection technique according to the following formulas [55]:

(1) $$SENSITIVITY\left[\%\right]=100\cdot \frac{TP}{TP+FN}$$

(2) $$SPECIFICITY\left[\%\right]=100\cdot \frac{TN}{TN+FP}$$

where FN indicates the “false negatives” (*i.e.*, pixels belonging to the HTN-containing layer that were not identified by the algorithm) and TN denotes the “true negatives” (*i.e.*, pixels not belonging to the target layer that were not colored by the algorithm). To further quantify the accuracy of our segmentation method, the DSC was also calculated for each frame according to the following formula [56,57]:

(3) $$DSC\left[\%\right]=100\cdot \frac{2TP}{2TP+FN+FP}$$

The values of each parameter were averaged over the considered sequence of 300 frames and then averaged again over the three samples realized for each concentration.

A different RULES configuration was finally determined for each tested HNT concentration employing each time a different subgroup of the 25 parameters available in the software, each one with its proper settings. The progressive optimization of the adopted RULES configuration always responded to the following criteria: (1) selective detection of the HNT-containing layer; (2) minimization of the FP appearance outside the HNT-containing layer; and (3) minimization of the FP appearance on the control phantom. In particular, in order to respect the third criterion, for each tested configuration, the initially calculated TP values were corrected by subtracting the number of pixels that were erroneously colored when applying the same configuration on a pure agarose control sample.

## 4. Conclusions

This study demonstrated the suitability of low-concentration HNTs as automatically detectable contrast agents for ultrasound targeted imaging at a conventional diagnostic frequency (10 MHz).

Characteristic “signatures” introduced by HNTs in the RF backscatter signals were experimentally identified and exploited to develop tailored color maps to be superimposed on conventional B-mode echographic images for automatic HNT detection, whose accuracy resulted in a sensitivity up to 60% coupled with a specificity above 95%. Among the investigated experimental conditions, the best HNT concentration for future medical applications resulted 1.5 mg/mL (equivalent to 33 × 10^10^ part/mL), which could represent a suitable concentration level also for the automatic echographic detection of different solid nanoparticles when employing a similar ultrasound frequency.

The clinical implementation of this technique, in conjunction with the development of specifically functionalized HNTs, will potentially result in tremendous diagnostic improvements related to the objective and non-ionizing identification of pathological tissues with cellular sensitivity.

Future studies will be devoted to the evaluation of HNT functionalization strategies and to the assessment of their performance as theranostic agents, capable of combining the diagnostic and therapeutic pathways in a single patient-personalized treatment, based on the selective and controlled release of drugs to the targeted diseased tissues.

## Figures and Tables

**Table 1:** Dice similarity coefficient (DSC) obtained through the automatic detection of HNTs for each considered concentration level.

HNT Concentration (10^10^ part/mL)	DSC (%)
5.5	4.3
16.5	19.1
33	67.3
66	49.9
